# Framing a New Nutrition Policy: Changes on Key Stakeholder’s Discourses throughout the Implementation of the Chilean Food Labelling Law

**DOI:** 10.3390/ijerph20095700

**Published:** 2023-05-01

**Authors:** Fernanda Mediano, Camila Fierro, Camila Corvalán, Marcela Reyes, Teresa Correa

**Affiliations:** 1 School of Psychology, Pontificia Universidad Católica de Chile, Santiago 8960040, Chile; mfmediano@uc.cl; 2 Carolina Population Center, University of North Carolina, Chapel Hill, NC 27516, USA; 3 School of Communication, Diego Portales University, Santiago 8370109, Chile; camila.fierro@mail.udp.cl; 4 Institute of Nutrition and Food Technology, University of Chile, Santiago 7830489, Chile; ccorvalan@inta.uchile.cl (C.C.); mreyes@inta.uchile.cl (M.R.)

**Keywords:** obesity prevention, food environment regulation, framing, food industry, Chile Labelling Law

## Abstract

The global implementation of structural policies to tackle obesity has been slow, likely because of the competing interests of governments and the food industry. We used the discussion of the Chilean Food Labeling Law to identify influential stakeholders in the media and their frames during different periods of the law’s implementation. This involved a content analysis of the food regulation media coverage in five key periods from 2007, when the food bill was first introduced in Congress, to 2018, when the second phase of the law was implemented (N = 1295). We found that most of the law coverage was through elite press. Half of the sources were from the food industry (26.7%) and government (26.2%), while other stakeholders, were less prevalent. Frames were mostly competing, except for cooperation with the law. The main food industry frame used during the discussion of the law was the “economic threat” (41.9%), whose prevalence decreased at the post-implementation period (13%, *p* < 0.01). No other relevant stakeholders changed their framing. Our results highlight that there are several aspects of public health communication, such as the type of media used, the involvement of scholars and civil society, and the framing, that could be improved to advance food environment policies.

## 1. Introduction

Health organizations are calling on countries to implement structural regulations to address the social, commercial, and environmental determinants of the population’s nutritional health [1,2,3,4]. Obesity and overweight have increased among children and adults worldwide, especially in Latin American countries [5], where almost 60% of adults [6] and more than 20% of minors aged 0–19 years are overweight or obese [7]. Driven by their high prevalence of overweight and obesity, several Latin American countries have implemented regulations to restrict the affordability, availability, and promotion of unhealthy food products, despite food industry opposition and several other barriers [8,9]. However, globally, the implementation of food policies is still low. It has been argued that the slow implementation of these regulations might be explained by political and economic interests led by powerful actors, such as the food industry [9] and political parties [10]. In this scenario, the media become powerful institutions where debates and disputes occur among stakeholders or interested parties [11]. Stakeholders that appear in the media as sources or spokespersons seek to influence the public discussion by framing the problem according to their approach. Frames define the problem, provide context, causal explanations, and suggest what is at stake through the selection, emphasis, and exclusion of keywords, metaphors, images, and sources [12]. 

The influence of media coverage both in the political agenda as well as in public opinion has been widely demonstrated in health policies [13,14,15]. Media coverage is a critical factor in the discussion and implementation of regulations as it may influence the regulations’ acceptance, the likelihood of being passed and enforced [16], and the effectiveness of such regulations [17]. In the case of debates about obesity and food laws, studies have found that official actors or stakeholders involved are the food industry, government, health organizations, consumer groups, academics, and medical researchers [10,18,19,20,21]. Often, the food industry and public health are seen as opposing groups that promote competing frames, resulting in a “framing contest” [22,23,24].

The food industry has been particularly successful in getting its frames covered by news stories [10,20,22,25]. The main frames used by the industry in response to food policy proposals include the acknowledgment of obesity as an issue and their willingness to change and be “part of the solution” [24,26,27,28] through self-regulation [26,27]. However, the industry often attacks the regulatory policies by stating that these policies would be ineffective [22], unsupported by evidence [27,29,30,31], and would imply negative economic consequences [9,25,31]. Another frame stresses the consumer’s responsibility and individual choice, or the nanny state frame, which argued that it is not the government’s place to dictate what individuals should consume [8,10,21,22,32]. In contrast, the government discourses emphasize consumer and public health protection [14], and the urgency of implementing policies to fight obesity, specially to protect children and vulnerable populations [22], which is aligned with the discourse of health organizations and experts [33]. 

Most studies on news media coverage of structural policies aimed at improving the food environments have focused on sugar-sweetened beverages (SSB) taxation, especially in countries outside Latin America [25,26,34,35]. Meanwhile, the media discussion about other food policies, such as marketing and labeling regulations have had less attention [10,21,22]. Additionally, only a few studies have analyzed changes in news framing before and after a food regulation has been implemented [19,25]. This study uses the case of Chile to fill this gap by analyzing the media coverage during the discussion and implementation of the Chilean food labeling and advertising law. 

Responding to Latin America’s high prevalence of malnutrition by excess of children and adults [36], and the evidence of the role of unhealthy food marketing on children’s preferences and diet, countries such as Brazil, Colombia, and Chile started working on advertising regulations to protect children [37]. As a result, effective in June 2016, the Chilean government implemented one of the most comprehensive regulations worldwide that had a multifactorial, innovative, structural approach [38]. The Food Labeling and Advertising Law (Law 20.606, henceforth “the Law”) combined different initiatives. It (1) requires front-of-package (FOP) warning labels (black stop signs) that identify pre-packaged foods and beverages that exceed established cutoffs of critical nutrients and become high in energy, saturated fats, sugars, and/or sodium (hereafter “high in” foods), (2) forbids “high in” foods in schools, and (3) restricts the marketing of “high in” foods to children under the age 14 years across different media platforms (television, radio, social media, radio, billboards, etc.) [38]. This law was implemented in three phases, with cutoffs becoming stricter in each stage: June 2016, June 2018, and June 2019. 

Although this law was first implemented in June 2016, it has a long story: the discussion in Congress started in 2007 and was approved in 2012 after several debates and a strong lobby from the food industry [37,39]. The proposal was initially motivated by the work of researchers led by an academic nutrition expert with global recognition (Dr. Ricardo Uauy) addressing the calls of the World Health Organization to implement policies to improve the population’s diet [40]. In the early 2000s, in Chile, 67% of adults and 46% of first-grade children presented excess weight [36,41,42]. As a result, the scientific community, legislators from all political parties and other policymakers discussed the need of a regulation. This was promoted by the Chilean Senate Health Committee, and in March 2007 a bill that sought to promote healthier food environments was presented in the Chilean National Congress. Despite the initial transversal political support, the Law was passed after five years of discussion (2012). Then, it required four more years to reach agreement on the implementation guidelines and enter into force (2016). In this process, aspects of the original proposal were removed or agreed to be further discussed in different bills [43,44]. 

The discussion of the law was particularly controversial because it was the first worldwide effort of implementing a front-of-package (FOP) message to discourage the consumption of unhealthy foods [43,45]. The introduction of the bill faced a strong opposition from the food industry [43,44,46,47], which was represented through different associations (e.g., Association of Food Companies in Chile (Chile Alimentos), Chile’s Manufacturer’s Association (SOFOFA), the Chilean Council for Self-regulation and Advertising Ethics (CONAR), and even an international organization, the Grocery Manufacturer Association [44,46]. In addition, in 2014, several big food companies, such as Coca-Cola, Nestlé, Carozzi, and Ferrero, even created an organization (AB Chile) to defend the interests of the industry [47]. Representatives of these associations as well as Chief Executive Officer (CEOs) of food companies had a strong presence in the media to warn the public and the government about the risks of the regulation [43].

Leading this initiative implied building evidence, developing alliances with different governmental institutions, as well as national and international health experts [48]. For instance, the Senate assembled two international summits on health and nutrition over the law discussion (2008, and 2011), which congregated national and international experts, academics and citizens to discuss the implications and feasibility of implementing this law [43]. In sum, the Law discussion convocated a variety of public and private stakeholders before it was finally implemented [44,46]. So far, several studies have evaluated the effectiveness of the law in TV advertising exposure, household purchases and people’s diet [49,50,51,52,53,54]. However, to date, no study has assessed the evolution of the stakeholders’ discourses about the Chilean regulation from its first discussions to the implementation. Therefore, an analysis about the media coverage of the sources and frames they promoted during the food regulation discussion and implementation could serve to understand the actors or stakeholders that had more exposure through the media, what their frames were, and, most importantly, whether and how they changed their approach after the law was implemented. This analysis can inform policymakers, academics, and advocacy groups from several countries that are discussing and implementing similar regulations [55,56].

Specifically, this study investigates the media coverage of the Chilean food regulation before and after its implementation covering five key periods that go from 2007, when the food bill was first introduced in Congress, to 2018, when the second phase of the law was implemented. We propose three research questions: 

RQ1: Who are the actors/stakeholders that had more influence through media exposure during the discussion and implementation of the Chilean food law? 

RQ2: What are the main frames used by the stakeholders during the discussion and implementation of the Chilean food law?

RQ3: How did the different stakeholders change their frames before and after the law was implemented? 

## 2. Materials and Methods

### 2.1. Sample 

We conducted quantitative content analyses of the main national media in Chile that covered the food regulation, including television, radio, newspaper, and online media (see Table 1 with the list of media). To select the news articles, we used the news clipping service NexNews, which is a database of published news in newspapers, television, radio, online news, and magazines from 2007 to the present. This clipping service has been used in other media coverage studies [57,58]. Before data collection, we identified five key periods during the discussion, approval, and implementation of the law, which lasted six months each:

(1) Initial discussion of the bill in the national Congress: March–August 2007
(2) Approval and promulgation of the regulation: April–September 2012
(3) Pre-implementation of the first phase of the regulation: January–June 2016
(4) Post-implementation of the first phase of the regulation: July–December 2016
(5) Implementation of the second phase of the regulation: April–September 2018

Within each of these five periods, we used the NexNews database to search news published in the Chilean media using the following search terms that include the different ways the bill was referred to by the press since its introduction: “Ley Superocho” (Supereight law, based on a national popular chocolate bar), “Ley Super ocho” (Super eight law), “Ley Super 8” (Super 8 law), “Ley semáforo” (Traffic light law), “Rotulación alimento/s” (Food/s labeling), “rotulado alimento/s” (food/s labels), “ley de alimento” (food/s law), ”ley de etiquetado” (label law), “sello + alimento” (label + food). 

Articles included in the sample had to include themes related to the food labeling and marketing regulation, such as the main restrictions of the law, arguments in support or opposition to the law, and expected outcomes. After cleaning the dataset and excluding non-related news articles, we obtained a total of 1295 news articles. 

### 2.2. Coding Process and Variables

The quantitative codebook was developed using inductive and deductive coding [59,60]. We first analyzed 100 news articles to identify the sources that were mentioned in the media as well as the frames promoted by each of them, i.e., inductive approach. Then, we developed a coding scheme based on the previous literature that investigated stakeholders’ discourses in food regulation processes, i.e., deductive approach, as well as the sources and news frames that were generated from the inductive phase. For example, the frame “nanny state” is based on previous literature while the frame “the law as an international benchmark” was extracted from the inductive analysis. 

Two trained coders analyzed a subsample of about 10% of the entire sample to test the instrument reliability. After refining and combining frames and providing more descriptions, coders achieved adequate levels of intercoder reliability (Cohen’s Kappa > 0.70). Then, five coders, all college-graduate journalists with previous experience in nutrition and health communication content analyses, were recruited and trained in three different sessions to complete the coding process. 

The news article was the unit of analysis. For each unit of analysis, coders identified the following codes: note date, type of media and its name, type of note, the first six sources mentioned in the news piece, and the frame or frames associated to each of the six sources identified source. 

Type of media: Media were classified as newspapers, television, online news media, radio, and magazines. The name of each media source was also recorded. 

Media main audience: The media were classified as elite media when the majority of their audience belonged to upper or middle-upper socioeconomic status based on the literature and reports [61,62]. 

Type of note: News were classified as news story, interview, column/letter to the editor, editorial, documentary/featured story, and other.

Stakeholders: To identify the main stakeholders, we coded the presence or absence of the first six sources mentioned in the news piece. The list of sources included: (1) Government authority, (2) Member of parliament, (3) Food industry, (4) Health organization, (5) Expert/scholar, (6) Spokesperson from schools/universities, i.e., teachers and administrators, (7) Consumer organizations, (8) Ex governmental authority, and (9) Others. (See Supplementary Materials Table S1 for a list of sources). 

News frames: The codebook included a list of 15 frames, which were coded as present or absent in the speech of each of the six sources identified in each news piece. The frames included: “labels are a source of information,” “Cooperation with the law,” “Solution to fight obesity,” “law is an international benchmark,” “Food industry does not look after children’s health,” “The law protects consumers and children,” “Nanny state,” “The law is confusing,” “The law is an economic threat to the food industry,” and “the law attempts against intellectual property,” “law enforcement,” “incentive/pressure to adapt,” “law is insufficient,” “the law as a step forward in public health policies,” and “lobby from the private sector” (see Supplementary Materials Table S2 for a list of frames with their description and examples). 

### 2.3. Analytical Strategy

We analyzed a total of 1295 news pieces. For each new piece, up to 5 sources with their respective frames were identified and coded. For general sample descriptions such as type of news media or type of news story we conducted descriptive statistics using the news piece as unit of analysis. Then, to analyze the total frequency of sources and frames, we added the number of sources and frames that appeared in each news piece. In these cases, we conducted descriptive statistics. To compare the stakeholder’s frames throughout the discussion of the law, we divided the entire sample into two main periods (pre-regulation vs. post-regulation) to increase the sample size of each period and be able to analyze the significant changes more clearly. The pre-regulation period contained the first three key periods (introduction of the bill (2007), approval/promulgation (2012), and pre-implementation of the first phase (January–June 2016). The post regulation period included post-implementation of the first phrase (July–December 2016) and implementation of the second phase (2018). To compare the frequency of frames pre- and post-regulation we run z-test test of proportions. 

## 3. Results

We found that most (n = 560 (43.2%) of the media coverage of the Chilean food regulation was in the first phase of the regulation implementation, followed by the pre-implementation period (n = 351 (27.1%)). In third place in term of coverage was the second phase of implementation (n = 242 (18.7%)). The regulation captures less media attention during the approval and promulgation discussion phase (n = 126 (9.7%)) and the initial discussion of the bill (n = 16 (1.2%)).

As shown in Table 1, we found that 30 different media covered the food labeling and advertising law over the five periods under study. Newspapers were the main media covering the regulation news (51.7%). Out of the 51.7% that comprised the newspaper coverage, three quarters of that newspaper coverage appeared in the press targeted to elites such as El Mercurio, La Tercera and the financial newspapers, such as Diario Financiero and El Pulso. Television and online media were less relevant (18.1% and 17.8%, respectively). Regarding the type of news covering the Chilean regulation, three fourths of the coverage appeared in news stories (75.9%). Less frequent were the interviews (10.9%), columns (8.0%), editorials (1.2%), letters to the editor (1.2%), and other (2.8%).

### 3.1. Actors/Stakeholders

Research question 1 asked about the actors/stakeholders that had more influence during the discussion and implementation of the food law. Half of the sources that appeared in the media belonged to the food industry (26.7%) and government authorities (26.2%), almost on equal parts. Less frequently covered were the experts/scholars (12.6%), congress people (9.6%) and health organizations (6.0%) (see Table 2). Further analyses showed that in the food industry, half of the sources come directly from big corporates, such as Carozzi (11.9%), McDonalds (10.8%), Ferrero (8.7%), Nestle (8.0%), and Coca-Cola (7.3%), and another 42% came from food organizations that represent big corporates such as Chile Alimentos, SOFOFA, and AB Chile, which was created to represent large food companies when the law was being discussed. Government sources covered corresponded mainly to health authorities (88%), and the rest included the presidency and other ministries, such as finance, social development, and education. The experts/scholars covered included national groups such as the Medical Association and academics. The international organizations covered were the WHO and PAHO. Among congress people, the senator Guido Girardi, one of the main drivers of the bill, had the greatest exposure through the media (See Table S1 for the list of sources).

### 3.2. Frames

Research question 2 asked about the main frames used by the stakeholders during the discussion and implementation of the food law. As shown in Table 3, in general, the most relevant frames during the discussion and implementation of the food law were the “law enforcement” (17.1%), which talked about standards of the new regulation and reports about food industry non-compliance. At the same time, the frame related to cooperation with the law was also prevalent (14.2%). This frame includes articles that talked about product reformulation in critical nutrients, such as saturated fats, sugar, sodium, and energy, to meet the new standards. It also incorporates coverage about food companies or supermarkets that included the labels before the law came into force or school vendors who adapted and began to sell fruits instead of sweet pastries. A few headlines that show this idea are the following: “Supermarkets seek to advance new food labeling by up to three months” and “The changes that will be implemented in schools to promote healthy snacks”. 

The third most relevant frame was the economic threat for the industry (11.6%). The food industry alerts about a “threat.” However, the sources do not specify the concrete impact for the industry. For example, the following headline illustrates the lack of specificity: Rodrigo Álvarez, president of AB Chile [the industry guild created during the law discussion]: “In this sector the economic impact will be very strong”. In a similar tone the president of Carozzi [a large chocolate, sweet and pasta company with branches throughout Latin America], Gonzalo Bofill, asserted: “The future is uncertain due to the implementation of improvised reforms.” Watts “warns about effects on costs due the advanced food labeling implementation”. The only story that talked about more concrete effects for the food industry was provided by Rodrigo Álvarez: “President of AB Chile: ‘They are close to US$50 million the stock of products that will be able to be sold (…). The problem is quite big, we have quantified about 50 million dollars in stock that will have problems with the commercialization from June 26th [because they would not have the labels on their packages].” Other discourses reproduced by the media during this period were that the labels were a source of information (8.3%), that the law sought to fight obesity (6.8%) and the pressures/incentives for the industry to adapt to the new standards (6/6%). The nanny state was not a prevalent frame in the media coverage. Only 1.1% of the coverage relied on that frame. 

#### 3.2.1. Frames by Key Stakeholders

A comparison of these frames by the three main stakeholders, i.e., food industry, government, and experts/scholars, revealed that the main frames promoted by the food industry were: cooperation with the law, economic threat, the law is confusing, and the law is insufficient. Meanwhile, the media promoted the following ideas coming from the government authorities: law enforcement, cooperation with the law, the labels are a source of information, the law seeks to fight obesity and the law as an international benchmark. The experts/scholars’ intervention revolved around the following ideas: the law is insufficient, the food labeling is a source of information, and the law is a solution to fight obesity (see Table 3). Although here we included the analysis of the three most covered stakeholders, it is worth mentioning that the legislator’s’ coverage (particularly senators) focused on the industry responsibility of the problem and denounced the lobbying from the private sector. 

#### 3.2.2. Changes on Key Stakeholders’ Discourses before and after the Law Implementation

Research question 3 asked how the different stakeholders changed their frames before and after the law was implemented. We compared the changes in frames of the three main stakeholders. As Table 4 shows, the food industry changed the strategy overtime. During the discussion of the law, the most important discourse for the food industry was the “economic threat”: 42% of the industry’s interventions used this frame. However, it significantly decreased to 13% after the law became effective (*p* < 0.01). At the same, they increased the discourse about the incentive/pressure to adapt as well (3.8–11.9%, *p* < 0.01) as the law being insufficient (8.3–14.9%, *p* < 0.01) and having an expropriation effect (0.3–5.5%, *p* < 0.01). The changes in the other frames were all non-significant. Although the government discourse was initially focused on the benefits of the regulation, and then on the relevance of the law enforcement, no significant differences were found before and after the regulation implementation (data not shown).

## 4. Discussion

This study analyzed how the media covered the discussion and implementation of the Chilean food labeling and advertising law, one of the most comprehensive nutrition regulations across the world [63]. The discussion of the regulation—from the initial introduction of the bill until it became effective—lasted about nine years, over three government administrations from the left and right wings. During this period, the media were powerful institutions where debates among stakeholders occurred and tried to influence the public discussion [43,46,47]. Therefore, by analyzing the media coverage, we aimed to understand the actors or stakeholders that had more exposure during the discussion and implementation of the law and how their frames changed before and after the law was implemented.

We found that most coverage appeared in newspapers, particularly targeted toward elites. The coverage was mainly through news stories rather than interviews, columns, or editorials. Despite this law affects people in general in their daily lives, it was still mainly covered through the elite press, which in Chile, targets the well-to-do sectors of society [64]. Eighty percent of the audience of the media that most covered the Law consisted of upper and middle-upper SES [61,62]. This shows that, for this food policy, the discussion was highly stratified [65], where not only powerful actors appeared more prominently in the coverage, but the discussion also occurred mainly in powerful news institutions. Given the economic concentration in Chile, the coverage of the discussion of the law was presumably guaranteed to influence the agenda [61]. 

The stakeholders that had greater influence in the discussion through the media were the food industry and the government. Half of the sources belonged to those two groups in equal parts. This is coherent with a study on the UK levy tax media coverage [25], where the industry and government were equally represented. We also found that stakeholders used the media to amplify their discourse, influencing the agenda. We found less coverage of the parliamentarians and health experts. When analyzing our findings in light of the story of this legislative process [43,46,47], it is possible to interpret that the discussion of the legislators in Congress was covered in the media mainly through the voice of the health authorities and the industry, representing the competing positions held by parliamentarians.

The sources covered from the government corresponded almost exclusively to health authorities, who were in charge of the implementation guidelines and enforcement of the regulation Other government authorities covered included the presidency and other ministries such as finance, social development, and education. We found that, in the food industry, half of the sources come from big corporates, such as Carozzi, McDonalds, Ferrero, Nestle, and Coca-Cola. The other important voice comes from industry and food companies’ associations. In the last period of the discussion AB Chile, the organization created in 2014 that represents the same big food corporations had wide media coverage. The voice of smaller food businesses was not prevalent in the discussion and implementation of the law, but the industry associations showed concern of the Law potential negative effects on small business [48]. Therefore, although most studies in the literature tend to study the food industry as a monolithic group and few studies have disentangled the analysis between larger and smaller companies [66], in this case, smaller companies were mostly absent from the media coverage and the discussion of the law. This fact reinforces the finding that most of the coverage focused on powerful actors by powerful media. Another interpretation is that they did not voice their concerns through the media because the regulation considered the needs of small businesses, providing more time and flexibility to adapt to the regulation requirements [47,67].

Scholars and experts were in third place of relevance. Our finding somewhat differs from the literature, which has found that the most relevant competing stakeholders have been the food industry and health organizations [23,24,30,68]. Despite less media exposure of experts and public health organizations in Chile, the discourses of government officials were built on the evidence provided by them [46,48,69]. The findings also show that members of the parliament, such as senators, appeared in the fourth place of coverage despite their relevance as proponents of the bill. Their less media coverage could be explained by the fact that they focused on other strategies, such as creating alliances with national and international health experts, which were key for the approval of the law [38,46,47].

We also found that civil society organizations such as consumer organizations were scantily present in the media coverage, which is similar to the experience in the UK [70]. The Chilean regulation was mainly pushed by academics and Congress members, rather than by civil society [52], which resonates with previous studies reporting low civil society involvement in food-related regulations in Latin America before the Chilean regulation was implemented [37]. This might also be related to the fact that civil society agreed with the proposed policy, as we found in the news analyzed in this study and the literature [47,48]. It is relevant to note that advocacy civil society groups in Latin America became more vocal after the Chilean law became effective and the Panamerican Health Organization (PAHO) implemented guidelines [71].

Regarding the frames represented in the Chilean media across the years, we found the most prominent frames were the law enforcement, cooperation with the law, and the regulation as an economic threat. We also found that government authorities maintained a consistent discourse over time about the relevance of consumers information, children health protection and regulation enforcement and cooperation. Other studies suggest that the health benefits of food regulations have also been a prominent frame used by governments proposing such policies [21,25,34], with a special emphasis on vulnerable populations [19,22]. The food industry used the “economic threat” as their principal frame to oppose to the food regulation, particularly when the law was being discussed in Congress but not yet approved. This aligns with research on stakeholders’ framings of food policies, such as SSBs taxation, school environment, and marketing regulations in countries, such as the UK, South Africa, and Mexico. The industry usually argues that these regulations would imply potential financial costs [22], economic harm [25], and industry loss of income and jobs [31]. 

During the period in which the industry’s main discourse about the law was the economic threat, the bill had several adaptations, reduced its scope and eliminated some aspects that were further discussed in a second food marketing law (20.869) [43,46,47,48]. Then, after the Law was passed, the industry also emphasized the difficulties of implementing the regulation, which resulted in the flexibilization of some restrictions and the extension of the preparation period before becoming effective [38,69]. The main adjustment was that the nutrient thresholds to determine “high-in” products would be implemented gradually, giving producers time to adjust their manufacturing processes. Additionally, the regulation included a longer adaptation for small businesses. More details can be found in the literature cited here, such as the work of Corvalan, Reyes [38], Corvalan, Reyes [43], Dorlach and Mertenskotter [44], Denecken and Schultz [46], Girardi G. [47], and Rodríguez Osiac, Cofré [69].

Once the policy was implemented, the industry changed the framing approach. It promoted the idea of being able to adapt to the new scenario, which is similar to the discourse of “being part of the solution” to fight obesity, a frame that the industry has used to avoid or delay food regulations elsewhere [27]. Moreover, the industry, in line with the government discourse, acknowledged the need for cooperation in this process. The change in the industry discourse might be explained by the fact that the economic harm frame has no supporting evidence [9]. In fact, a study that evaluated the impact of the Chilean law on labor market outcomes in the food industry found that neither employment nor real wages were affected by the regulation [66]. 

Contrary to previous evidence [10,21,22], the “nanny state” frame, which criticizes these public policies because they hamper people’s freedom and choices, was not relevant in the Chilean media coverage. It is possible that this frame was avoided in Chile given that food labelling policies are basically based on providing information rather than restricting any kind of liberty. We also believe it might have been relevant the fact that the regulation was aimed to protect children, considered a vulnerable population, and that the context was one of high obesity prevalence and burden, even compared to other countries in the region [48].

It is important to note that despite the long period of discussion, the Chilean food labeling and advertising law was fully implemented in 2019. Moreover, the available evidence shows the industry has mostly complied with the policy [49,50,51,52]. Although the present study describes the regulation media coverage and cannot identify its impact on the discussion and implementation process, we interpret the consistent discourse of health authorities as a key factor that allowed the discussion to continue despite the strong resistance of the industry and finance sectors. This aligns with the studies of the story of the law which propose that Chilean bureaucrats defended the legality of the regulation by emphasizing the priority of public health over economic concerns and national regulatory autonomy [44], and that the alliance between the academia and policymakers was the most important factor in passing the Chilean Law [46,48]. Additionally, the industry frames presented by the media acknowledged the need to address this public health problem, which might also have contributed to continuing the discussion of the bill, as discussed in other studies [48]. However, the industry lobbying also covered by the media resulted both in delaying the implementation of the regulation and in an opportunity to negotiate some aspects of the regulation [38,46,48]. It must be noticed that a small proportion of news coverage (3.2%) showed that the government authorities also used the economic threat frame to provide support to the food industry [48]. This reflects that the legislative process happened during three governments, one of them from the right wing, when the authorities used these economic frames [48]. However, as it has been described in the literature, the long discussion of the Law and its regulation implied a negotiation of regulatory aspects but did not imply modifying the central goals of the law [44,46,48].

From this analysis, we believe countries on ongoing regulatory discussions might try to move the policies forward by building alliances and disseminating (i) data on the local and global obesity issue, (ii) a solid body of evidence supporting the regulation, (iii) evidence of the lack of negative externalities for the food sector, and the country economy in general, and (iv) a set of strategies and actions that allow the industry’s adaptation to the new regulation. Additionally, we consider including the voice of civil society in the media could have helped the policy, given that the literature describes that the civil society agreed with the government taking actions to protect children and the population’s health [47]. 

The present study is not exempt from limitations. The news analyzed in this study were drawn from the NexNews database which, despite covering a wide variety of Chilean media, might have missed news covered by smaller and local media. This study relies on traditional media content to understand not only how the press covered the discussion and implementation of the food law, but also how the most powerful stakeholders negotiated their influence and frames to shape the public discussion towards the law. Although the media are still powerful institutions where the debates among stakeholders occur, future research should also analyze how the stakeholders are using social media, such as Twitter, Instagram, or YouTube, to promote and negotiate their approaches in the discussion of a relevant policy like this one. It must be noted that this content analysis reports on the stakeholders and frames of news covering the food regulation discussion, but it is not able to report on the audience reached by that news coverage. Future studies could evaluate exposure to the news frames and the potential impact of these messages on the audience [17]. Additionally, future research should also include interviews or focus groups with the stakeholders to understand the rationale behind their approaches and strategies.

## 5. Conclusions

The media coverage during the discussion and implementation of the Chilean food labeling and advertising law was highly stratified as the discussion occurred mainly in powerful news institutions and covered the most powerful stakeholders. Our findings suggest that the wide coverage of the industry’s economic harm discourse may have contributed to delaying the regulation and negotiating some restrictions in favor of the industry’s adaptation. However, the consistent and evidence-based discourse of the health authorities might have contributed to passing a Law that conserved its core elements. We also observed that both the government and the industry shared the frame of collaboration, which might have contributed to the dialogue across years. 

The regulation discussion was less covered by the popular media, and the civil society was barely covered in the media discussion. Given that the evidence shows that media coverage can influence public opinion and the acceptance of health policies, it is essential to think how this type of initiative is communicated to people outside the elite, and how these groups could be incorporated into the media discussion more effectively.

## Figures and Tables

**Table 1:** Chilean Media Covering the food regulation 2007–2018.

Media	Media Name	N (1295)	Percentage (%)
Total Newspapers		670	51.7
	El Mercurio	178	26.6
	La Tercera	116	17.3
	Diario Financiero	114	17.0
	Pulso	75	11.2
	La Cuarta	40	6.0
	La Segunda	38	5.7
	Publimetro	34	5.1
	Las Últimas Noticias	33	4.9
	La Hora	23	3.4
	HoyXHoy	19	2.8
Total TV		235	18.1
	CNN	87	37.0
	24 Horas	58	24.7
	Chilevisión	26	11.1
	Mega	24	10.2
	TVN	22	9.4
	Canal 13	18	7.7
Online News Media		230	17.8
	Cooperativa.cl	60	26.1
	Radio Biobío.cl	44	19.1
	Emol	39	17.0
	La Tercera Online	29	12.6
	El Mostrador	26	11.3
	Agricultura Online	16	7.0
	El Dínamo	10	4.3
	The Clinic Online	6	2.6
Total Radio		159	12.3
	Radio BioBío	45	28.3
	Radio Cooperativa	36	22.6
	ADN Radio	36	22.6
	Radio Agricultura	23	14.5
	Tele13 Radio	19	11.9
Magazines		1	0.1

**Table 2:** Actors/stakeholders that appear in the media covering the Chilean food regulation.

Actors/Stakeholders	Total (N = 2964)	%
Food industry	790	26.7
Government authority	777	26.2
Expert/scholar	374	12.6
Member of parliament	283	9.6
Health organization	179	6.0
Schools/universities	94	3.2
Consumer organizations	72	2.4
Former Govt authority	17	0.6
Other	378	12.8

**Table 3:** Frames and discourses about the Chilean food law 2007–2018 in total and by the three main stakeholders covered in the media.

Frames	Total		Food Industry		Govt Authority		Expert/Scholar	
	N (2422)	%	N (737)	%	N (661)	%	N (234)	%
Law enforcement	415	17.1	25	3.4	242	36.6	20	8.5
Cooperation with the law	343	14.2	205	27.8	68	10.3	11	4.7
Law as an economic threat	282	11.6	187	25.4	21	3.2	8	3.4
Food labeling as a source of information	201	8.3	11	1.5	77	11.6	39	16.7
Solution to fight obesity	164	6.8	4	0.5	70	10.6	26	11.1
Incentive/pressure to adapt	160	6.6	62	8.4	39	5.9	22	9.4
Law is confusing	178	7.3	121	16.4	5	0.8	17	7.3
Law is insufficient	288	11.9	89	12.1	14	2.1	50	21.4
Law as an international benchmark	108	4.5	1	0.1	48	7.3	5	2.1
Industry does not look after children’s health	70	2.9	3	0.4	8	1.2	8	3.4
The law protects consumers and children	68	2.8	0	0.0	31	4.7	12	5.1
The law as a step forward in public health policies	67	2.8	2	0.3	35	5.3	10	4.3
Nanny State	27	1.1	2	0.3	0	0	4	1.7
Lobby from the private sector	18	0.7	1	0.1	1	0.2	1	0.4
The law has an expropriation character	33	1.4	24	3.3	2	0.3	1	0.4

**Table 4:** Frames of the food industry pre-regulation and post-regulation.

	Pre-Regulation		Post-Regulation		
Frame	N (315)	%	N (422)	%	z-Test
Law enforcement	10	3.2	15	3.6	n.s.
Cooperation with the law	80	25.4	125	29.6	n.s.
Law as an economic threat	132	41.9	55	13.0	*p* < 0.001
Food labeling as a source of information	4	1.3	7	1.7	n.s.
Solution to fight obesity	1	0.3	3	0.7	n.s.
Incentive/pressure to adapt	12	3.8	50	11.8	*p* < 0.001
Law is confusing	45	14.3	76	18.0	n.s.
Law is insufficient	26	8.3	63	14.9	*p* < 0.01
Law as an international benchmark	0	0.0	1	0.2	n.s.
Industry does not look after children’s health	0	0.0	3	0.7	n.s.
The law protects consumers and children	0	0.0	0	0.0	n.s.
The law as a step forward in public health policies	1	0.3	1	0.2	n.s.
Nanny State	2	0.6	0	0.0	n.s.
Lobby from the private sector	1	0.3	0	0.0	n.s.
The law has an expropriatory character	1	0.3	23	5.5	*p* < 0.001

## Data Availability

The datasets used and/or analyzed during the current study are available from the corresponding author on reasonable request.

## Supplementary Materials

The following supporting information can be downloaded at: https://www.mdpi.com/article/10.3390/ijerph20095700/s1, Table S1: Description of frames and discourses about the Chilean food law. Table S2. List of stakeholders and sources.
