# Supplementary material for: Framing a New Nutrition Policy: Changes on Key Stakeholder’s Discourses throughout the Implementation of the Chilean Food Labelling Law
Source: Int J Environ Res Public Health. 2023 May 1;20(9):5700. doi: 10.3390/ijerph20095700 (PMC10178804; doi:10.3390/ijerph20095700)
Supplement: Supplementary file 1 [file ijerph-20-05700-s001.zip › ijerph-2242568-supplementary.pdf]

Table S1: Description of frames and discourses about the Chilean food law.

Frames	Descriptions and examples
Law enforcement	Sources talking about the control and surveillance actions, calls to comply with the law, cases of the industry noncompliance.<br>Example: Minster Carmen Castillo announced that those who do not respect the labeling will be fined and closed.
Cooperation with the law	Sources assert that the industry or schools are adopting the necessary changes to comply with law.<br>Examples: From Chilealimentos (an industry association): "The industry will always be available to contribute with the solution"<br>School vendor asserted that the municipality has trained her to orient her business toward healthier products and alternatives.
Food labeling as a source of information	Sources assert that the role of this initiative is to provide information to the consumers so at the time of the purchase they make an informed decision.
Solution to fight obesity	Sources assert that with the food labeling law, it will be possible to combat obesity rates and change people's eating habits. They also may assert that there is evidence that the labeling law will have results.<br>Example: "It is necessary to promote drastic measures to promote healthy life and discard bad eating habits."
Industry does not look after children's health	Sources accuse the food industry of going against children's rights in favor of their economic interests.<br>Example: Senator Girardi accused brands like Pepsi of being "21st century's pedophiles who violate children's human rights."
Law as an international benchmark	Sources emphasize that thanks to this law Chile is positioned as an international leader in this type of public policy.<br>Example: "Authorities assure that the Chilean model is being studied by other countries"
Law as an economic threat	Sources emphasize the costs and economic losses that this law generates in industries and small businesses.<br>Examples:<br>"Loss of US$50 million in products that cannot be sold to the lack of labels," "Complications with imports/exports," Juan José Llugany from Carozzi: "Much imported products will stop arriving because international brands will not be willing to put a different label on it."
Law is insufficient	The sources raise objections and suggestions to improve the law because it alone will not end obesity given that it is a multifactorial problem. In addition, they assert that obesity is fought with education not with the imposition of a law.<br>Example: Chilealimentos (industry association): Given that obesity is a multifactorial problem, it is important to target the rest of the causes such as nutrition education and sedentary lifestyles. Otherwise, this initiative will not achieve the expected objectives. Principal of Instituto Nacional (a renowned public high school): "More than a prohibitive law, we must promote education."
Law is confusing	Sources state that the new law generates confusion and uncertainty for the industry and producers regarding when the enforcements begin, when the labels should be applied.<br>Example:<br>AB Chile (an industry association): This generates enormous uncertainty (for the industry). The lack of certainty required by any production or legal process is striking."
Incentive/pressure to adapt	Sources assert that the law is an incentive/pressure for the industry to adapt its products to avoid the labels, contribute to healthy eating and change people's habits.<br>Example: Incentive to reformulate the products.

The law protects consumers and children	Sources state that the objective of the law is to protect consumers and children from bad eating habits or from "misleading advertising."
The law as a step forward in public health policies	Sources assert that this law is a positive advance or first step to improve the country's public policies related to obesity and demonstrates a commitment towards the health of the Chileans.
Nanny State	The sources' discourses focus on the fact that the law damages consumers' individual freedom and choices. Example: El Mercurio's op ed: "(The law) presumes that the state has the ability to define what is best for the members of society" Carlos Larraín (a right-wing politician): "The state had interfered in many areas, but it had not done in digestion."
Lobby from the private sector	Sources accuse that there has been lobbying and negotiations with the private sector in order to stop the labeling law or making the regulation more flexible. Example: Senator Girardi accused lobbying from the private sector with the Ministry of Economy to intervene the law.
The law has an expropriatory character	Sources assert that the law is an attack to intellectual property" by having to change the packaging conditions given that they cannot longer carry cartoons. Example: Chile killed the Tiger Tony.

Table S2. List of stakeholders and sources.

Stackeholders		Sources
Gubernamental authority	Health authority	-Ministry of Health<br>-Superintendence of Health<br>-Public Health Institute<br>- Choose to Live Healthy Program. Social Development Ministry.
	Other gubernamental authority	-Ministry of Education<br>-National School Meal Program. Ministry of Education.<br>-Ministry of Economy/Finance<br>- National Institute of Industrial Property. Finance Ministry.<br>-Ministry of Social Development
	Presidency	-Presidency
Member of Parliament	Senate	-Senator Guido Girardi
Industries	Associations related to the food industry	-Food and Beverage Association<br>-Chilean Federation of Industry (Sofofa).<br>-Chilefood<br>-Refreshing Drinks Association<br>-The Grocery Manufacturers Association<br>-National Confederation of micro and small-medium companies (Conapyme).<br>-Supermarkets Association<br>-The Santiago Chamber of Commerce<br>- Chile's Suppliers Association
	Associations related to the defense of freedom of commercial expression, self-regulation and advertising ethics.	-National Association of Advertisers<br>-Promoplan Agency
	Companies related to the food industry	-Carozzi, Soprole, PepsiCo, McDonalds, Nestle, Iansa, Colun, Lider-Walmart, Unilever, Dos en Uno, Evercrisp, Coca Cola, Fruna, Ferrero, Ambrosoli, Jumbo, Unimarc, Santa Isabel, Tottus.
Health related organizations	National	-Medical College<br>-College of Nutritionists<br>- National confederation of health municipal employees (Confusam).<br>- Global Alliance Against Obesity (Chile)
	International	-WHO<br>-FAO<br>-PAHO
Experts	Academia	Institute of Nutrition and Food Technology
Schools/Universities	Authority of School´s institution<br>Parents<br>School kiosk owner	
Consumer-oriented organizations	National Corporation of Consumers and Users of Chile<br>Organization of Consumers and Users<br>National Consumer Service	
